# Computed tomography imaging and clinical features of congenital hepatoblastoma: A retrospective analysis

**DOI:** 10.1097/MD.0000000000021174

**Published:** 2020-07-31

**Authors:** Li Li, Wen Liu, Rong Wen, Ke Jin

**Affiliations:** a Department of Radiology, Hunan Children's Hospital, University of South China; b Department of Radiology, The Third Xiangya Hospital of Central South University; c Department of Pathology, Hunan Children's Hospital, University of South China, Changsha, Hunan, China.

**Keywords:** alpha-fetoprotein, computed tomography, congenital hepatoblastoma, neonates

## Abstract

Congenital hepatoblastoma (CHB) is the most common hepatic malignant tumor of fetus or neonates, but few studies focusing on the radiological characteristics of CHB have been reported to date.

To investigate the characteristic clinical and computed tomography (CT) findings of CHB to facilitate recognition and noninvasive diagnosis.

Medical records of 7 patients with CHB were retrospectively reviewed. The demographic, clinical, and laboratory data were extracted from the electronic medical records. Two pediatric radiologists evaluated the abdominal CT examinations for the hepatic tumor location, size, enhancement characteristics, vascular invasion, and intra-/extra-hepatic metastasis.

Among the included 7 patients (3 males and 4 females), only 1 had an elevated serum alpha-fetoprotein level. All patients had solitary intrahepatic mass with a mean size of 4.7 cm (range: 2.9–10.2 cm), of which liver SV-VII were most involved. 4/7 tumors were round while 3/7 irregular or lobulated. 6/7 tumors were well-defined. Microhemorrhage, cystic necrosis, and coarse calcification were present in 5/7, 4/7, and 1/7 tumors, respectively. All lesions showed inhomogeneously significant enhancement, with multiple nodular or striped appearance in the center and periphery of the tumors on the arterial phase, and then the enhancement area showed progressive expansion and fusion filling over time but the attenuation gradually declined on the portal and delayed phases, and finally the majority (6/7) of tumors presented multiple band- or island-like characteristics with prominently peripheral enhancement on the delayed phase while the remaining 1 relatively small tumor showed nearly complete but inhomogenous enhancement. In addition, only 1/7 tumor had hilar hepatic bile duct and portal vein invasion and secondary intra-hepatic bile duct dilation. No metastatic lesions were identified in all patients at diagnosis. The abdominal aorta distal to the coeliac trunk was significantly narrowed in 3/7 patients. Pathological examinations suggested that 6/7 tumors showed fetal histology with only 1 containing mesenchymal elements.

The relationship between serum alpha-fetoprotein and CHB could be more complicated and yet to be determined. Dynamic contrast-enhanced CT can facilitate recognition and noninvasive diagnosis of CHB, presenting a pattern of progressive expansion and fusion filling but inhomogeneously significant enhancement.

## 1 Introduction

Hepatoblastoma (HB) is the most common hepatic malignant tumor of childhood, mostly occurred before 5 years of age. Congenital HB (CHB), those detected in utero or diagnosed within the first 28 days after birth, accounts for less than 10% of all pediatric HB.^[1–4]^ Imaging examinations play a vital role in managing pediatric liver tumors, which can be highly suggestive of a specific diagnosis based on sufficient clinical information. Up to now, however, few studies focusing on the radiological characteristics of CHB has been reported.^[5]^ It has not been studied as thoroughly as HB diagnosed at an older age.

Therefore, the purpose of our study was to review the characteristic clinical and radiologic findings of 7 patients with CHB to facilitate noninvasively preoperative diagnosis and avoid misdiagnosis or unnecessary surgical exploration.

## 2 Materials and methods

This retrospective study was approved by our institutional review board and patient consent was waived. We identified 7 HB patients diagnosed in the neonatal period based on pathological findings from 2012 to 2019 at our hospital through queries of the radiology and pathology search engines. Patient demographic information, clinical signs and symptoms, laboratory tests, and treatment were obtained from the electronic medical records. All patients underwent abdominal contrast-enhanced computed tomography (CT) examinations before surgical resection. Two pediatric radiologists (L. L., with 5 years of experience, and K.J., with 20 years of experience) reviewed CT scans of the eligible patients by consensus, and evaluated the main imaging features regarding hepatic tumor location, size, morphology, margin, enhancement pattern, presence or absence of cystic necrosis, hemorrhage or calcifications, adjacent anatomical structure involvement, and intra-/extra-hepatic metastasis. The tumor size was indicated by the maximum dimension. The radiologists were blinded to the clinical information and pathological diagnosis.

## 3 Results

The demographic and clinical characteristics of included patients in this study are shown in Table 1. All the patients (3 males and 4 females) were born at term, with a median age of 13.5 days (range 2–24 days) at diagnosis. Only 1 patient presented with elevated preoperative alpha-fetoprotein (AFP) of 178539.8 ng/mL, and the remaining 6 patients had normal AFP levels (normal range for neonates: 100∼10^5^ng/mL^6^). Other blood tests including hemoglobin, white blood cells count, and platelet level were relatively nonspecific. Focal abnormality in the liver was detected by ultrasound in 4 patients antenatally, while the liver tumors were found accidentally in the other 3 patients due to respiratory diseases. No patients had a family history of Beckwith–Wiedemann syndrome, hemihypertrophy, or familial polyposis coli.

**Table 1:**
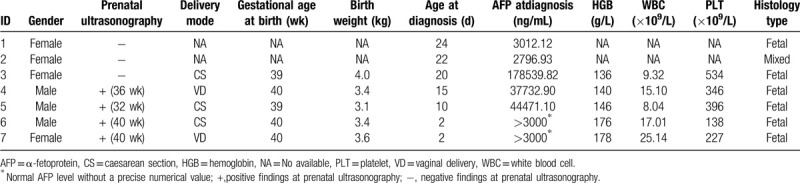
Demographic and clinical characteristics of patients at admission.

**Figure 1:**
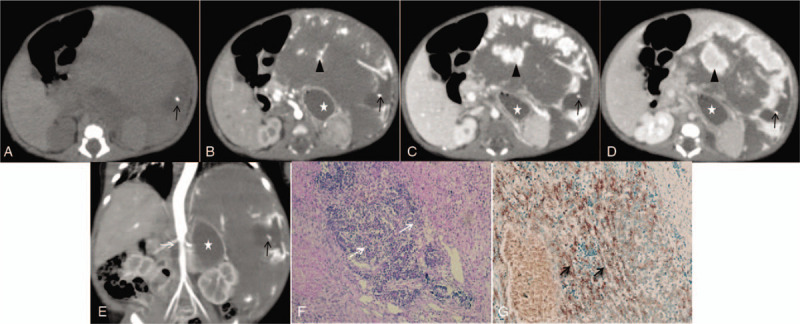
A 10-day-old female neonate with giant exogenous CHB in the left lobe of liver (segments II and III), with a maximum diameter of 10.3 cm. A. Small nodular calcification within the patchy cystic necrosis area (black arrow) in the mass was seen on the unenhanced CT; B. There were multiple nodular/ striped distinct enhancement (black triangle) in the center and periphery of the mass on the arterial phase, and gastric cavity (white star) and spleen were compressed and moved inside; C, D. The enhancement area of the mass showed progressive expansion and fusion filling over time but the attenuation gradually declined (black triangle) on the portal and delayed phases, and finally achieved multiple band- and island-like characteristics. No enhancement was seen in the cystic necrosis area (black arrow). E. The abdominal aorta distal to the coeliac trunk was significantly narrowed (white arrow) on the coronal plane. F. HE staining showed double-layered cord-like or adenoid-like structure (white arrow), accompanied by vascular regeneration and fibroplasia (H&E × 100); **G**. Immunohistochemical staining showed positive expression of Glypican in the tumor cells (black arrow). CHB = congenital hepatoblastoma, CT = computed tomography.

**Figure 2:**
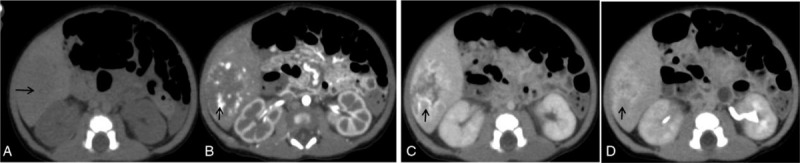
A 2-day-old female neonate with CHB in liver segment SVI, with a maximum diameter of 4.4 cm. A. Small patchy slightly high-density microhemorrhages (black arrow)in the mass was indistinctly seen on the unenhanced CT; B. There were multiple nodular distinct enhancement (black arrow) in the center and periphery of the mass on the arterial phase. C, D. The enhancement of the mass presented progressive expansion and fusion filling (black arrow) on the portal and delayed phases, and finally achieved nearly complete but inhomogeneous enhancement. CHB = congenital hepatoblastoma, CT = computed tomography.

At the abdominal CT examinations, all the 7 cases of CHB were single solid and the mean tumor size was 4.7 cm (range: 2.9–10.2 cm). Liver S (V∼VII) were most involved, with 2 cases in S VI, 2 in S VII, 1 in S (V + VI), 1in S (II + III), and 1 in S (IVa + V + VIII). The majority (6/7) of tumors were well-circumscribed. 4/7 tumors presented round while 3/7 tumors with extrahepatic extension irregular or lobulated. Microhemorrhage, cystic necrosis, and nodular calcification was seen in 5/7, 4/7, and 1/7 tumors, respectively. All tumors showed inhomogeneously significant enhancement. All tumors presented marked enhancement with multiple nodular or striped appearance in the center and periphery of the tumors on the arterial phase. The enhancement area showed progressive expansion and fusion filling over time but the attenuation gradually declined on the portal and delayed phases, and finally the majority (6/7) of tumors presented multiple band- or island-like characteristics with prominently peripheral enhancement on the delayed phase (Fig. 1) while the remaining 1 relatively small tumor showed nearly complete but inhomogenous enhancement (Fig. 2). No enhancement was seen in the necrotic area. In addition, only 1/7 tumor had hilar hepatic bile duct and portal vein invasion and secondary intra-hepatic bile duct dilation. The abdominal aorta distal to the coeliac trunk was significantly narrowed in 3/7 patients. No metastatic lesions were identified in all patients at diagnosis.

Pathologically, 6/7 tumors revealed fetal histology with only 1/7 containing mesenchymal elements. Under microscopic examination, the tumor cells were arranged in cord-like pattern and abundant tumor vessels and blood sinuses were present in the stroma. In addition, most tumors contained multifocal extramedullary hematopoiesis. Focal or patchy hemorrhage and necrosis were detected in 6 tumors and calcification in 1. Immunohistochemical staining showed positive expression of Ki-67 in 7 cases, glypican in 5 cases, AFP in 1 case, cytokeratin in 5 cases, vimentin in 3 cases and CD34 in 3 cases.

## 4 Discussion

Most neonatal hepatic neoplasms present as an asymptomatic abdominal mass detected on routine ultrasound,^[7]^ and CHB is also no exception. Thus, the diagnosis might be delayed because of the indolent and nonspecific presentation, resulting in less than 10% of HB diagnosed in the neonatal period.^[2,3]^ There were only eligible 7 CHB cases with pathological confirmation, with a slightly female predominance, which was inconsistent with previous reports^[4]^ mainly due to the relatively small sample size. Moreover, none of the patients in our study had any known risk factors associated with increased incidence of HB,^[4]^ including genetic factors, such as Beckwith Weidemann Syndrome, familial adenomatous polyposis, low birth weight or prematurity, eclampsia or severe pre-eclampsia during pregnancy, and maternal smoking.

Serum AFP has been found to be an useful marker for HB screening.^[3,8]^ However, only 1 patient had an elevated AFP in our series, which did not seem to show a significant association of AFP level with CHB. It is noteworthy that AFP level is normally high at birth and may vary widely among individuals during the neonatal period,^[6,8,9]^ and then decrease rapidly between 6 and 12 months of age, when it attains adult status.^[10]^ Hence, it is a challenge to distinguish tumoral secretion from physiological elevated AFP concentration solely based on 1-time measurement in neonates, which tend to result in a false-negative result.^[7,11,12]^ For this reason, active surveillance of AFP is of great clinical significance for suspected neonates, and CHB should be considered when AFP level increases over time after birth in term neonates and needs to be confirmed by further needle biopsy or resectionas necessary.^[13]^

From the perspective of pathological origin, HB can be classified into 2 main types:

(1) epithelial;
(2) mixed epithelial and mesenchymal.^[14]^

The former is further divided into multiple subtypes, including fetal, embryonal, small cell undifferentiated, mixed epithelial, and cholangioblastic, according to the degree of cellular differentiation. CHB mainly consists of fetal and embryonal components compared with infants beyond the neonatal period.^[3]^ Patients with pure well-differentiated fetal HB usually have a good outcome with complete excision and no chemotherapy.^[14–16]^ 6/7 tumors in our study showed fetal subtype with only 1 containing mesenchymal elements and all patients had a favorable outcome after excision without additional chemotherapy. The histological immunomarkers that were detected in our series included Ki-67, glypican, alpha-fetoprotein (AFP), cytokeratin, vimentin and B-lymphocyte marker CD34. In recent years, more and more attention has been paid to the significance of glypican-3 in the diagnosis, occurrence and development of HB. Glypican-3 is highly expressed in fetal epithelium, weakly expressed or absent in embryonic epithelial cells, but not expressed in small cell tumor cells.^[17]^ In routine immunohistochemical detection of HB, the intensity and sensitivity of glypican-3 is higher than that of AFP and it has been reported to be an excellent marker for the diagnosis of infantile HB.^[18]^

Imaging can play a crucial role in diagnosis of hepatic neoplasms in neonates. Most CHB developed in the right lobe of the liver probably resulting from the difference of the circulation to the right and left lobes in fetus. 1/7 tumor among our cases appeared calcification. Calcification is relatively rare and presents usually small and fine in epithelial tumors.^[14,19,20]^ Consistent with the observations seen in previous literature,^[21]^ we also found the occurrence of hemorrhage and cystic necrosis in CHB, and higher incidences in larger tumors. On dynamic contrast-enhanced CT, all the 7 tumors showed early peripheral and intratumoral nodular or striped significant enhancement on the arterial phase, and then the enhancement area showed progressive expansion and fusion filling over time but the attenuation gradually declined, presenting multiple band-like or island-like characteristics with prominently peripheral enhancement on the portal and delayed phases. Our imaging finds validated other 3 previous studys which also discribed hypervascular HB.^[5,22,23]^ This imaging appearrence differs from that of HB in older children generally showing enhance less than surrounding normal liver.^[21,24]^ We infered these imaging features of CHB might be associated with tumor microanatomy that was highly vascularized reflected in abundant tumor vessels and large blood sinuses in the stroma^[22,25]^ and intratumorally multifocal extramedullary hematopoiesis.^[26]^ Decreased aortic caliber below the celiac axis may be present when there is enlargement of the celiac axis or hepatic artery due to vascular shunting into the hepatic mass.^[5,23]^

The most challenging differential diagnosis of CHB is congenital hemangioma. A single AFP measurement is often unhelpful because the normal range at birth is high. Although congenital hemangioma has some distinctive imaging manifestations—a heterogeneous mass with no pseudocapsule, more common calcification, rarely extrahepatic extension, and gradual centripetal fill-in enhanced pattern till delayed phase, the best strategy to distinguish these 2 hepatic neoplasms is a short period of observation. The congenital hemangioma size may decrease and serum AFP level often falls rapidly after birth,^[13]^ while CHB dose not. In our study, several patients were misdiagnosed as congenital hemangioma at admission, which might result from our lacking recognition of CT characteristics of CHB and neglecting the importance of serial measurements of AFP in the neonatal period.

The main limitations of our study were the retrospective nature and a small sample size, though to our knowledge this is the largest case series reported regarding the CT characteristics of CHB. In addition, it didn’t allow us to adequately understand the imaging difference between different histologic types given that our study subjects were mainly fetal type.

Our study suggested that the clinical and CT characteristics of CHB could differ from those occurred beyond the neonatal age. AFP level should be interpreted with more caution in neonates, and most CHB had fetal components in histopathology. Dynamic contrast-enhanced CT can facilitate recognition and noninvasive diagnosis of CHB, presenting a pattern of progressive expansion and fusion filling but inhomogeneously significant enhancement. However, the estimates of our study are based on too few patients to reach a firmer conclusion. Larger multi-center studies confirming these radiological findings are needed.

## Author contributions

**Conceptualization:** Li Li, Wen Liu, Ke Jin.

**Data creation:** Li Li, Wen Liu, Rong Wen, Ke Jin. **Formal analysis:** Li Li, Wen Liu.

**Methodology:** Li Li.

**Supervision:** Li Li, Ke Jin, Wen Liu, Rong Wen. **Validation:** Li Li, Ke Jin.

**Writing – original draft:** Li Li.

**Writing – review & editing:** Li Li, Wen Liu, Rong Wen, Ke Jin.
